# Trends in incidence and associated risk factors of suicide mortality in patients with non‐small cell lung cancer

**DOI:** 10.1002/cam4.1656

**Published:** 2018-07-03

**Authors:** Huaqiang Zhou, Wei Xian, Yaxiong Zhang, Gang Chen, Shen Zhao, Xi Chen, Zhonghan Zhang, Jiayi Shen, Shaodong Hong, Yan Huang, Li Zhang

**Affiliations:** ^1^ Department of Medical Oncology Sun Yat‐sen University Cancer Center Guangzhou China; ^2^ State Key Laboratory of Oncology in South China Guangzhou China; ^3^ Collaborative Innovation Center for Cancer Medicine Guangzhou China; ^4^ Zhongshan School of Medicine Sun Yat‐sen University Guangzhou China

**Keywords:** non‐small cell lung carcinoma, prognosis, risk factors, SEER, suicide

## Abstract

Lung cancer patients have an increased risk for committing suicide. But no comprehensive study about the suicide issues among non‐small‐cell lung cancer (NSCLC) patients has been published. We aimed to estimate the trend of suicide rate and identify the high‐risk group of NSCLC patients. Patients diagnosed with primary NSCLC were identified from Surveillance, Epidemiology, and End Results (SEER) database (1973‐2013). Suicide mortality rate (SMR) were calculated. Multivariable logistic regression was employed to find out independent risk factors for suicide. Among 495 889 NSCLC patients, 694 (0.14%) of them died from suicide. The suicide mortality rates have significantly decreased (before 1993: 0.21%, 1994‐2003: 0.16%, after 2004: 0.09%, *P *<* *.001). Male (OR 6.22, 95% CI: 4.96‐7.98, *P *<* *.001), white (OR 3.89, 95% CI: 2.66‐5.97, *P *<* *.001), being unmarried (OR 1.43, 95% CI: 1.22‐1.67, *P *<* *.001), the elderly (60‐74 vs <60: OR 1.24, 95% CI: 1.03‐1.50, *P *=* *.024, >75 vs <60: OR 1.31, 95% CI: 1.05‐1.63, *P *=* *.018) were independently associated with higher risk of suicide mortality. Surgery (OR: 1.44, 95% CI: 1.19‐1.73, *P *<* *.001) was also relative with higher risk of suicide. Our study observed significant decrease in suicide mortality among NSCLC patients in US over past decades. Older age, male sex, unmarried status, and surgery were risk factors of committing suicide. Clinicians should be aware of these high‐risk groups.

## Abstract 2

There is significant decrease in suicide mortality among NSCLC patients in US over past decades. Older age, male sex, unmarried status, surgery were risk factors of committing suicide.

## 1 INTRODUCTION

Suicide is one of major causes of non‐cancer‐related death, which took up 1.4% of all deaths worldwide in 2015.^1^ Several studies have also demonstrated that the suicide rate of cancer patients is twice that of general population.^2^, ^3^, ^4^, ^5^, ^6^ Notably, when considering different anatomic cancer sites, patients diagnosed with lung cancer had a higher suicide rate than those with other cancer, with a standardized mortality ratio of 5.74.^2^ Factors associated with increased suicide risk among lung cancer patients were Asians, men, older, widowed, small cell lung carcinoma, metastatic, and refusing treatment.^7^

Lung cancer is second most common cancer, and 85% of them are non‐small cell lung carcinoma (NSCLC).^8^ Although several researchers have observed a high risk of suicide among lung cancer patients (being discussed as a single cancer entity), further examinations of patients with the most common subtype of lung cancer (NSCLC) are required, because of totally different distribution, treatment strategy and prognosis between subtypes.^2^, ^7^, ^9^ However, to our knowledge, a comprehensive study about the suicide issues among NSCLC patients has not been specifically published. Given that potential suicide prevention, knowing the trend of suicide rate and the high‐risk patient is of great importance. Therefore, we conducted this study using a large population‐based database to estimate the trend of suicide rate and identify the high‐risk group of NSCLC patients. In addition, we also performed a sub‐analysis of patients diagnosed from 2004 to 2013 to depict recent issues.

## 2 MATERIAL AND METHODS

National Cancer Institute's Surveillance, Epidemiology, and End Results (SEER) database is an authoritative source of information on cancer incidence and survival in the United States. SEER database encompasses about 28% of United State population and collect cases diagnosed between 1973 and 2013.

We extracted data of patients diagnosed with primary NSCLC from SEER database(1973‐2013) using the SEER*Stat software (v8.3.4, Cancer Statistic Branch, NCI, Calverton), using International Classification of Disease for Oncology, Third Edition (ICD‐O‐3), morphology codes: 8012/3, 8046/3, 8070/3, 8140/3, 8240/3, 8250/3, 8560/3 and 9053/3; and site codes: C33.9, C34.0, C34.1, C34.2, C34.3, C34.8 and C34.9.^10^, ^11^ Patients with unknown follow‐up, diagnosed below 18 and recognized by autopsy and death certificate were excluded.

Patients whose cause of death variable coded as “Suicide and self‐inflicted injury” were identified. We obtained the demographic and clinicopathological data from the SEER database, including age, sex, race, marital status, year at diagnosis, state, tumor site, grade, histologic type, stage, surgery, cause of death, survival time, vital status and radiation. Patients were divided into 3 groups according to age at diagnosis (younger than 60 years, 60‐74 years, and older than 75 years). Race were sorted by white, black and others. We classified patients as married or unmarried. Year of diagnosis were separated into 3 groups (before 1993, 1994‐2003, after 2004). We classified the tumor site as upper lung, middle lung, lower lung, and bronchus/others. Grade of tumor were categorized into I/II, III/IV, and unknown groups. Surgery and radiation were both classified as performed, not performed, and unknown. Disease stage for the analysis was coded based on the variable “SEER Historic Stage A.” We give a value of 0.5 months to those who didn't survive for a full month after diagnosis, because SEER record their survival time in months.

Univariate analysis using chi‐square test was used to compare patients committed suicide with those died from other causes. Multivariable logistic regression was employed to find out independent risk factors for suicide. All statistical analyses were performed using R version 3.4.2 software (Institute for Statistics and Mathematics, Vienna, Austria; http://www.r-project.org). Statistical significance was set at two‐sided *P *<* *.05.

## 3 RESULTS

### 3.1 Patient cohort characteristics

In total, 495 889 patients diagnosed with nonsmall‐cell lung cancer were extracted. Among all patients, 694 (0.14%) of them died from suicide. Among all patients, 207 306 (41.8%) of them are female while 288 583 (58.2%) of them are male. Among those patients committed suicide, 77 (11.1%) of them are female, and 617 (88.9%) of them are male. In total, 403 288 (81.3%) of them are white, 59 005 (11.9%) of them are black, and 33 596 (6.8%) of them are unknown and other races. As for those suicided patients, 634 (91.4%) of them are white, 25 (3.6%) of them are black, and 35 (5.0%) of them are unknown or other races. In all, 138 210 (27.9%) of them were diagnosed below 60, 237 648 (47.9%) of them were diagnosed between 60 to 75, and 120 031 (24.2%) of them were diagnosed over 75.

### 3.2 Differences in rates of suicide mortality by decade and state

The suicide mortality rates between all 3 time intervals were significantly different (before 1993: 0.21%, 1994‐2003: 0.16%, after 2004: 0.09%, overall: 0.14%, *P *<* *.001) (Table 1). Patients from Kentucky, Louisiana, New Jersey were not recorded before 1993. When considering differences among different time intervals, California (before 1993: 0.25%, 1994‐2003: 0.18%, after 2004: 0.10%, *P *<* *.001), Michigan (before 1993: 0.19%, 1994‐2003: 0.12%, after 2004: 0.07%, *P *=* *.001), Washington (before 1993: 0.27%, 1994‐2003:0.19%, after 2004: 0.11%, *P *=* *.010) show significant drop of suicide mortality rate.

**Table 1:** Suicide mortality rates by states and time of diagnosis

State	Before 1993	SMR (%)	1994‐2003	SMR (%)	After 2004	SMR (%)	Total	SMR (%)	*P*
All states	246	0.21	227	0.16	221	0.09	694	0.14	<.001
California	66	0.25	92	0.18	84	0.10	242	0.15	<.001
Connecticut	12	0.11	9	0.08	5	0.04	26	0.08	.004
Georgia	24	0.28	31	0.22	32	0.11	87	0.17	.015
Hawaii	6	0.14	6	0.20	5	0.12	17	0.15	.100
Iowa	25	0.15	16	0.17	11	0.10	52	0.14	<.001
Kentucky	—	—	6	0.09	15	0.07	21	0.08	.058
Louisiana	—	—	5	0.07	14	0.09	19	0.08	.048
Michigan	48	0.19	17	0.12	10	0.07	75	0.14	<.001
New Jersey	—	—	7	0.06	16	0.06	23	0.06	.068
New Mexico	10	0.20	13	0.37	9	0.21	32	0.25	.017
Utah	13	0.42	6	0.29	6	0.22	25	0.32	.014
Washington	42	0.27	19	0.19	14	0.11	75	0.20	<.001

SMR, Suicide mortality rate.

### 3.3 Risk factors of suicide mortality in the entire cohort

Univariate analysis showed that suicide mortality was significantly higher in male patient (*P *<* *.001), white (*P* < .001), diagnosed between 60 and 75 (*P *=* *.034), squamous cell carcinoma (*P *=* *.006), surgery (*P *<* *.001) and without radiation therapy (*P *=* *.018) (Table 2).

Multivariate logistic regression was then performed including factors significant on univariate analysis. In terms of demographic factors, sex (male vs female: OR 6.22, 95% CI : 4.96‐7.98, *P *<* *.001), race (white vs black: OR 3.89, 95% CI: 2.66‐5.97, *P *<* *.001, unknown/others vs black: OR 2.75, 95%CI: 1.65‐4.66, *P *<* *.001), marital status (unmarried vs married: OR 1.43, 95% CI: 1.22‐1.67, *P *<* *.001), year at diagnosis (‐1993 vs 2004+: OR 1.83, 95% CI: 1.43‐2.34, *P *<* *.001,1994‐2003 vs 2004+: OR 1.61, 95% CI: 1.34‐1.95, *P *<* *.001), age at diagnosis (60‐74 vs <60: OR 1.24, 95% CI: 1.03‐1.50, *P *=* *.022, >75 vs <60: OR 1.32, 95% CI: 1.06‐1.64, *P *=* *.014) were independently associated with higher risk of suicide mortality. As for clinical factors, surgery (Yes vs No: OR: 1.44, 95% CI: 1.19‐1.73, *P *<* *.001) was relative to higher risk of suicide (Table 2).

**Table 2:** Results of univariate analysis and multivariable logistic regression for the entire cohort

Characteristics	Overall	Suicide	SMR (%)	*P* a	OR	95% CI	*P* b
n	495 889	694	0.14				
Sex							
Female	207 306	77	0.04	<.001	1.00		
Male	288 583	617	0.21		6.22	4.92‐7.98	<.001
Race							
Black	59 005	25	0.04	<.001	1.00		
Unknown/others	33 596	35	0.10		2.75	1.65‐4.66	<.001
White	403 288	634	0.16		3.89	2.66‐5.97	<.001
Marital							
Unmarried	223 335	299	0.13	.319	1.43	1.22‐1.67	<.001
Married	272 554	395	0.14		1.00		
Year at diagnosis							
2004‐	236 391	221	0.09	<.001	1.00		
‐1993	116 202	246	0.21		1.83	1.43‐2.34	<.001
1994‐2003	143 296	227	0.16		1.61	1.34‐1.95	<.001
Age at diagnosis							
<60	138 210	164	0.12	.034	1.00		
60‐75	237 648	360	0.15		1.24	1.03‐1.50	.024
>75	120 031	170	0.14		1.31	1.05‐1.63	.018
Site							
Upper	250 535	347	0.14	.495			
Bronchus/other	107 101	144	0.13				
Middle	20 688	24	0.12				
Lower	117 565	179	0.15				
Grade							
I/II	100 566	144	0.14	.175			
III/IV	178 591	270	0.15				
Unknown	216 732	280	0.13				
Histology							
BAC	16 275	19	0.12	.006	1.00		
LCC	33 119	48	0.14		1.12	0.66‐1.96	.679
Others	70 826	71	0.10		1.18	0.72‐2.03	.533
S	149 388	245	0.16		1.21	0.77‐2.01	.431
AC	218 088	296	0.14		1.35	0.87‐2.24	.206
ASC	8193	15	0.18		1.45	0.72‐2.85	.286
Stage							
Distant	224 941	246	0.11	<.001	1.00		
Localized	73 293	111	0.15		1.15	0.89‐1.48	.284
Regional	109 710	149	0.14		1.05	0.84‐1.30	.684
Unstaged	87 945	188	0.21		1.17	0.91‐1.52	.219
Surgery							
No	364 393	444	0.12	<.001	1.00		
Unknown	1081	1	0.09		0.78	0.04‐3.45	.801
Yes	130 415	249	0.19		1.44	1.19‐1.73	<.001
Months							
≥6 years	46 144	68	0.15	.762			
1 year	310 902	436	0.14				
2 years	74 528	111	0.15				
3 years	33 462	38	0.11				
4 years	18 465	26	0.14				
5 years	12 388	15	0.12				
Radiation							
No	265 814	397	0.15	.018	1.00		
Unknown	2932	8	0.27		1.66	0.75‐3.13	.158
Yes	227 143	289	0.13		0.88	0.74‐1.03	.109

AC, Adenocarcinoma; ASC, Adenosquamous carcinoma; BAC, Bronchioloalveolar; CI, confidence interval; LCC, Large cell carcinoma; S, Squamous; SMR, Suicide mortality rate.

a *P* value on univariate analysis.

b *P* value on logistic regression.

### 3.4 Sub‐analysis of patients diagnosed from 2004 to 2013

This subgroup of patients can better represent the demographic and clinicopathological character of recent patients. So we do the sub‐analysis of patients diagnosed from 2004 to 2013.

Univariate analysis displayed that higher suicide mortality rate was associated with male patients (*P *<* *.001), white patients (*P *<* *.001), and patients didn't have radiation therapy (*P *=* *.115). Concerning the time after diagnosis, the highest suicide mortality rate was found to be the first year after diagnosis (*P *=* *.008).

Multivariate logistic regression was operated considering factors significant on univariate analysis. In respect of demographic factors, sex (male vs female: OR 7.12, 95% CI: 4.77‐11.12, *P *<* *.001), race (white vs black: OR 4.76, 95% CI: 2.41‐11.23, *P *<* *.001, unknown/others vs black: OR 2.46, 95% CI: 0.94‐6.82, *P *=* *.069), marital status (unmarried vs married: OR 1.41, 95% CI:1.08‐1.85, *P *=* *.012) were independently correlated to higher risk of suicide mortality. As for clinical factors, radiation (Yes vs No: OR:0.74, 95% CI: 0.55‐0.99, *P *=* *.046) were relative to higher risk of suicide. Finally, time elapsed from cancer diagnosis was also relative with higher rate of suicide mortality (*P *=* *.008), with the first year of diagnosis taking the highest rate (OR 4.79, 95% CI: 1.93‐15.97, *P *=* *.003), followed by the second year (OR 4.31, 95% CI: 1.68‐14.60, *P *=* *.007) (Table 3).

**Table 3:** Results of univariate analysis and multivariable logistic regression for patients diagnosed 2004‐2013

Characteristics	Overall	Suicide	SMR (%)	*P* a	OR	95% CI	*P* b
n	236 391	221	0.09				
Sex							
Male	128 081	196	0.15	<.001	7.12	4.77‐11.12	<.001
Female	108 310	25	0.02		1.00		
Race (%)							
Black	28 908	7	0.02	<.001	1.00		
Unknown/others	18 072	10	0.06		2.46	0.94‐6.82	.069
White	189 411	204	0.11		4.76	2.41‐11.23	<.001
Marital							
Unmarried	115 326	109	0.09	.927	1.41	1.08‐1.85	.012
Married	121 065	112	0.09		1.00		
Age at diagnosis							
<60	61 248	48	0.08	.221	1.00		
60‐75	109 508	102	0.09		1.08	0.77‐1.54	.676
>75	65 635	71	0.11		1.22	0.84‐1.79	.295
Site							
Upper	121 665	108	0.09	.328	1.00		
Bronchus/other	45 091	39	0.09		0.92	0.62‐1.33	.66
Middle	10 070	7	0.07		0.84	0.35‐1.67	.653
Lower	59 565	67	0.11		1.24	0.91‐1.68	.163
Grade							
I/II	50 487	41	0.08	.468	1.00		
III/IV	72 850	75	0.10		1.22	0.83‐1.82	.316
Unknown	113 054	105	0.09		1.22	0.82‐1.83	.338
Histology							
BAC	5805	1	0.02	.292			
LCC	6269	6	0.10				
Others	49 618	43	0.09				
S	61 110	58	0.09				
AC	110 006	107	0.10				
ASC	3583	6	0.17				
Stage							
Distant	132 310	122	0.09	.977	1.00		
Localized	41 332	40	0.10		1.24	0.8‐1.89	.321
Regional	56 549	54	0.10		1.10	0.76‐1.56	.608
Unstaged	6200	5	0.08		0.86	0.3‐1.92	.751
Surgery							
No	184 415	170	0.09	.761	1.00		
Unknown	560	1	0.18		2.06	0.12‐9.27	.475
Yes	51 416	50	0.10		1.34	0.88‐2.04	.168
Months							
≥6 years	15 970	4	0.03	.008	1.00		
1 year	147 889	159	0.11		4.79	1.93‐15.97	.003
2 years	37 135	35	0.09		4.31	1.68‐14.6	.007
3 years	18 001	13	0.07		3.24	1.14‐11.58	.041
4 years	10 353	8	0.08		3.32	1.04‐12.46	.051
5 years	7043	2	0.03		1.18	0.16‐6.06	.848
Radiation							
No	136 621	143	0.10	.115	1.00		
Unknown	1329	1	0.08		0.63	0.04‐2.83	.65
Yes	98 441	77	0.08		0.74	0.55‐0.99	.046

AC: Adenocarcinoma; ASC: Adenosquamous carcinoma; BAC: Bronchioloalveolar; CI: confidence interval; LCC: Large cell carcinoma; S: Squamous; SMR: Suicide mortality rate.

a *P* value on univariate analysis.

b *P* value on logistic regression.

## 4 DISCUSSION

Our study observed significant improvement in suicide prevention among NSCLC patients in US over past decades. Urban et al^9^ found that suicide has not changed significantly decreased in lung cancer over time. However, in contrast to rising suicide rate of US general population, the suicide mortality rate of NSCLC patients has decreased considerably over past decades, which is consistent with previous study about suicide trend among cancer patients.^12^, ^13^, ^14^ This result may be associated with relatively better prognosis of NSCLC, because of early screening test for lung cancer and significant advances in treatment, such as chemotherapy and targeted therapy.^15^, ^16^, ^17^

Demographic characteristics associated with an increased rate of suicide in the NSCLC patients, such as older age, male sex, race were similar to those in general population.^18^ Earlier research showed that older people tend to commit complete suicide among general population.^19^, ^20^ Older patients with cancer are also high‐risk group, which is consistent with our research.^21^, ^22^, ^23^ Older patients usually encountered with greater disease burden, and social psychological pressure. Higher suicide rate of older NSCLC patients may be related to pressure and depression.^20^ Another possible reason is that, they hold the rational will of ending their life at the right time.^24^ Male sex is a risk factor of suicide in NSCLC patients, and it is accordant with trends in general population and those with other cancer.^2^, ^25^ Although depression seemed to be higher in female patients with NSCLC, male patients are more likely to succeed in ending their own life.^26^, ^27^ However, the incidence of female suicidal behavior in NSCLC patients may be underrepresented, because failed suicide attempts were not recorded in the SEER database.^22^ Race has a significant impact on suicidal ideation. The risk of dying from suicide was more than double for the white NSCLC patients than for the black patients.^28^ The reason is for higher suicide rate in white patients with NSCLC is still unknown, and hopelessness in those patients is likely to associate with suicidal behavior.^29^ In addition, unmarried NSCLC patients are easier exposed to suicide attempts. Married patients have a greater socioeconomic status than unmarried.^30^, ^31^ Many cancer research studies have reported a poor prognosis in unmarried patients.^32^, ^33^, ^34^

Interestingly, characteristics of NSCLC seems to be not relevant to suicide of patients, which is controversial with former analysis.^9^ In patients diagnosed between 1973 and 2013, clinical characteristics such as primary site, histologic type, historic stage have no significant influence on suicide. The same as those diagnosed recently. A possible reason is that multiple primary tumor may interfere with the result. In our study, patients with multiple primary tumor were excluded to prevent interference of other tumors. Another possible reason is that the prognosis of patients with advanced NSCLC is poor, they may die from the disease itself rather than other causes. Patients diagnosed between 1973 and 2013 who undergo treatment like surgery are more likely to suicide. This is also different from previous research.^9^ It can be explained by debility and loss of autonomy brought by the curative surgery toward NSCLC.^35^

When considering patients diagnosed between 1973 and 2013, Suicide mortality rate (SMR) is not significantly associated with time after diagnosed. But SMR is observed to be higher within the first year of NSCLC diagnosis among patients diagnosed between 2004 and 2013. It has been reported that cancer patients were at high risk of suicide within the first year of diagnosis and associated demographic and clinical factors were analyzed.^36^ The different result can be possibly explained by the improvement of life quality of long‐term survivor of NSCLC patients, and the major reason for suicidal behavior of recent NSCLC patients is possibly shock of cancer diagnosis.^37^, ^38^

While the demographic risk factors of cancer‐related suicide are focused on, psychological and social risk factors are frequently missed. Suicide is a complicated phenomenon that biological, psychological and social risk factors would interact and influence on it. Cancer diagnosis may lead to demoralization of NSCLC patients, such as hopelessness and helplessness, which can lead to suicidal ideation.^39^ Poor consequences of cancer treatment may bring physical and mental pain to NSCLC patients.^40^ It has been reported that cancer patients with low socioeconomic status and family support are more likely to suicide.^41^ The National Comprehensive Cancer Network provides a distress management guideline that recommends screening all patients for distress.^42^ Our findings may assist oncologists to effectively identify those NSCLC patients at higher risk of suicide, specifically for older, white, unmarried male patients with surgery. For those NSCLC patients with high risk of suicide, we should pay more attention, because appropriate psychosocial interventions have a positive impact on quality of life.^43^ To reduce cancer‐related suicide, patients’ understanding of cancer diagnosis and treatment options should be ensured.^44^ Besides, promoting family communication combine with encouraging self‐determination and participation in treatment can mitigate social risk of suicide.^44^, ^45^

There are some limitations in our study. Suicide is a complicated phenomenon affected by factors such as economic level and education level. These factors are not included in SEER database. Additionally, SEER database only contains data of US patients, so our study is limited to US patients, and research over the world is still needed. Moreover, details of treatment to NSCLC were not taken in, and we only know whether patients have undergone surgery and radiation or not. Details of treatment such as the time after surgery and chemotherapy may be associated with suicide of cancer patient.^3^ Furthermore, our study can't obtain suicide attempts data of NSCLC patients, and patients potential to suicide were likely to be underestimated.

## 5 CONCLUSIONS

In summary, our study observed a significant decrease in suicide mortality among NSCLC patients in US over past decades. Older age, male sex, unmarried status, and surgery were risk factors of committing suicide. Grade, stage, histologic type and primary site of NSCLC appear not relate to suicide. It can help clinicians identified these NSCLC patients for better support and suicide prevention. Further studies are still needed.

## CONFLICT OF INTEREST

All of the authors have no conflicts of interested to declare.
